# One pedigree we all may have come from – did Adam and Eve have the chromosome 2 fusion?

**DOI:** 10.1186/s13039-016-0283-3

**Published:** 2016-09-26

**Authors:** Paweł Stankiewicz

**Affiliations:** Department of Molecular and Human Genetics, Baylor College of Medicine, One Baylor Plaza, Rm ABBR-R809, Houston, TX 77030 USA

**Keywords:** Chromosomal fusion, Genomic rearrangements, Homozygosity, Human evolution, Gene loss, Evolutionary advantage

## Abstract

**Background:**

In contrast to Great Apes, who have 48 chromosomes, modern humans and likely Neandertals and Denisovans have and had, respectively, 46 chromosomes. The reduction in chromosome number was caused by the head-to-head fusion of two ancestral chromosomes to form human chromosome 2 (HSA2) and may have contributed to the reproductive barrier with Great Apes.

**Results:**

Next generation sequencing and molecular clock analyses estimated that this fusion arose prior to our last common ancestor with Neandertal and Denisovan hominins ~ 0.74 - 4.5 million years ago.

**Hypotheses:**

I propose that, unlike recurrent Robertsonian translocations in humans, the HSA2 fusion was a single nonrecurrent event that spread through a small polygamous clan population bottleneck. Its heterozygous to homozygous conversion, fixation, and accumulation in the succeeding populations was likely facilitated by an evolutionary advantage through the genomic loss rather than deregulation of expression of the gene(s) flanking the HSA2 fusion site at 2q13.

**Conclusions:**

The origin of HSA2 might have been a critical evolutionary event influencing higher cognitive functions in various early subspecies of hominins. Next generation sequencing of *Homo heidelbergensis* and *Homo erectus* genomes and complete reconstruction of DNA sequence of the orthologous subtelomeric chromosomes in Great Apes should enable more precise timing of HSA2 formation and better understanding of its evolutionary consequences.

## Background

Approximately one dozen of microscopically visible chromosomal aberrations, multiple subtelomeric heterochromatin expansions, and hundreds of submicroscopic structural variants involving both coding and non-coding sequences distinguish human and Great Ape genomes [1, 2]. Among these numerous rearrangements, there are only two evolutionary chromosome translocations, t(4;19) in *Gorilla gorilla* [3–7] and head-to-head fusion forming human chromosome 2 (HSA2) [4, 8–10]. The latter reduced the number of chromosomes from 48 in Great Apes to 46 in humans.

### Mechanism of HSA2 formation

**Fig. 1:**
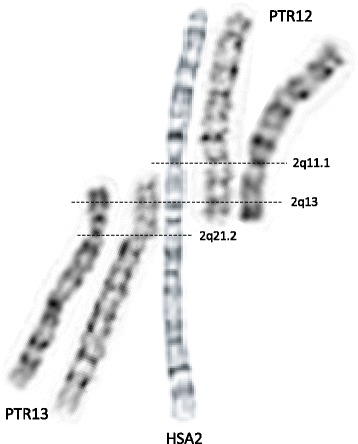
Alignment of G-banded human (HSA2) and chimpanzee (PTR12 and PTR13) metaphase chromosomes demonstrates that a few megabases of subtelomeric regions (mainly repetitive satellite DNA) are absent in the human genome. DNA seqeunece of these genomic regions in PTR12 and PTR13 is not well annotated and their gene content is essentially unknown. Note that this sequence may not represent the deleted fragments of the ancestral chromosomes IIp and IIq [16]

The HSA2 fusion site was first mapped using FISH within the telomeric repetitive sequences corresponding to the short arms of orthologous chimpanzee chromosomes PTR12 and PTR13 [11, 12]. Fan et al. analyzed the low-copy repeat (LCR)-rich complex genomic structure and gene content of the ~ 600 kb region surrounding the fusion site at 2q13 and mapped the chromosome junction within degenerate telomeric arrays in an intron of the non-coding *DDX11L2* (DEAD/H-box helicase 11 like 2) transcript [13, 14]. Of note, the fusion site is resistant to PCR amplification and cloning. Genome-wide comparisons of recent chimpanzee and human LCRs revealed a 40 kb genomic fragment that map to four regions in the human genome, but is represented 400 times (‘hyperexpansion’) within the chimpanzee genome [15]. It was proposed that amplification of this interval occurred prior to HSA2 fusion and LCRs located close to the ancestral fusion sites might have facilitated the rearrangement between the closely flanking telomeric repeats of the two ancestral chromosomes. More recently, Ventura et al. [16] proposed a molecular-evolutionary model in which an ancestral human chimpanzee pericentric inversion and HSA2 fusion both predisposed and protected the chimpanzee and human genomes, respectively, to the formation of subtelomeric heterochromatin. Importantly, chromosome alignments, including positions of the active and vestigial [17] centromeres, as well as the current genome assemblies of HSA2, PTR12, and PTR13 chromosomes indicate that a few megabases of mostly repetitive satellite-rich subtelomeric DNA have been lost on both ancestral chromosomes (referred to as IIp and IIq) during the formation of HSA2 (Fig. 1).

### Time of HSA2 origin

Computational genomic comparisons of AT to GC (weak-to-strong) substitutions between the human and chimpanzee genomes using rhesus macaque as a reference allowed estimation of the fusion date at 0.74 million years ago (Mya) with a 95 % confidence interval 0–2.81 Mya [18]. In support of this notion, using next generation sequencing (NGS) with a high-coverage (30x), Meyer et al. [19] have reconstructed a genome of a Denisovan, an extinct relative of Neandertals. They identified the HSA2 junction and concluded that Denisovans (and presumably Neandertals) shared the fused chromosome 2 with modern humans. Corroboratively, Ventura et al. [16] estimated that HSA2 fusion must have occurred early (>4 Mya) during evolution, and most recently, Miga [20] analyzed satellite DNA in the HSA2 pseudocentromere at 2q21.2 [17] and calculated HSA2 fusion at ~ 3.5 Mya (with a range of ~ 2.5 - 4.5 Mya).

## Hypotheses

### Formation of HSA2 was a nonrecurrent rearrangement event

**Fig. 2:**
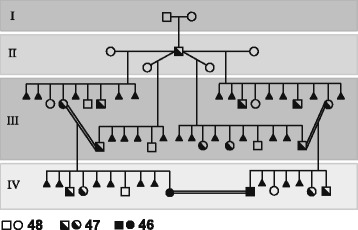
A suggested gorilla-like [46] polygamous pedigree of a putative early modern human clan in which HSA2 was transmitted, implemented, and its heterozygous status converted to homozygous. A male carrier of a *de novo* heterozygous HSA2 (half filled square, IInd generation), had multiple children (IIIrd generation) with a few female partners. Analogous to carriers of Robertsonian translocations, he is expected to have had healthy progeny with balanced 48 (blank square/circle) and 47 (half filled square/circle) chromosomes in addition to multiple miscarriages due to chromosomal imbalances (filled triangles). Note that the unions between individuals with 47 chromosomes heterozygous for HSA2 likely produced healthy children with balanced 48 and 47 chromosomes as well as heathy subjects with 46 chromosomes (homozygous for HSA2, IVth generation). The latter individuals are expected to have had an unaffected fecundity when inbreeding with carriers of balanced 46 chromosomes homozygous for HSA2. The evolutionary advantage might have resulted from an enhanced fertility e.g. due to testis-expressed *DDX11L2*. Alternatively, assortative mating between individuals with higher cognitive functions due to genomic loss (heterozygous or homozygous) of gene(s) important for brain development or function might have facilitated the successful spreading of HSA2

(1) Given the high structural complexity of the genomic region flanking the HSA2 fusion site on chromosome 2q13 and the fact that no HSA2 junction polymorphisms have been identified in modern humans, it is very unlikely that HSA2 fusion was a recurrent genomic rearrangement. I propose that HSA2 arose only once likely in one early modern human male and was subsequently transmitted and accumulated as a heterozygous event, converted to the homozygous state due to inbreeding in a small “bottleneck” polygamous clan (Fig. 2), and spread in the succeeding populations.

### Evolutionary advantage due to the genomic loss

(2) The most comprehensive comparative analyses of the human and chimpanzee transcriptomes of 21,000 protein-coding genes active in brain, heart, liver, kidney, and testis revealed that the patterns of tissue-specific gene expression and gene sequences are markedly similar [21]. I hypothesize that heterozygous and homozygous genomic loss of the putative gene(s) embedded in the repetitive subtelomeric sequences might have played a more important evolutionary role than deregulation of gene expression and/or a change of function of the genes flanking the HSA2 fusion site.

## Discussion

### Recurrent chromosomal translocations

Robertsonian translocations (ROBs), resulting from meiotic fusion of two acrocentric chromosomes (13–15, 21, or 22), occur in approximately one in every 1000 newborns. Although most ROB carriers are healthy, they are at increased risk of infertility and spontaneous abortions due to transmission of the unbalanced gametes [22]. ROBs are formed likely due to nonallelic homologous recombination (NAHR) between long stretches of highly similar repetitive satellite DNA sequences in the short arms of acrocentric chromosomes. Numerous examples of various ROB fusion sites have been documented in the literature [23–25].

To date, only a few recurrent non-Robertsonian constitutional translocations have been described in humans. The most frequent t(11;22)(q23;q11.2) results from a rearrangement between palindromic AT-rich repeats (PATRRs) (cruciform structures) [26, 27]. PATRRs were also found to mediate other recurrent constitutional translocations: t(17;22)(q11.2;q11.2) [28], t(4;22)(q35.1;q11.2) [29], t(8;22)(q24.13;q11.21) [30], and t(3;8)(p14.2;q24.1 [31]. In addition, NAHR between inter-chromosomal LCRs (segmental duplications) was shown to result in recurrent constitutional translocations: t(4;8)(p16.2;p23.1), t(4;11)(p16.2;p15.4), and t(8;12)(p23.1;p13.31) [32, 33]. Similarly to ROBs, various recombination sites have been found in the NAHR-mediating LCRs.

Interestingly, breakpoints of the well-known human recurrent pericentric inversion inv(2)(p11.2q13) have been mapped ~ 3 Mb proximal to the HSA2 fusion site [34, 35]. The less frequent fragile site (FRA2B) at 2q13, which has not been fine mapped thus far, may have an impact on the reuse of inv(2)(p11.2q13) and might have influenced the formation of the HSA2 fusion [36].

### Homozygosity for constitutional chromosomal aberrations

Very rarely, chromosome aberrations are found in a homozygous state [37, 38]. Thus far, only 13 families with Robertsonian translocation present in a homozygous state (44 chromosomes) have been reported [39–51]. Recently, Song et al. [51] described a healthy 44,XY,der(14;15)(q10;q10),der(14;15)(q10;q10) male with no apparent defects in spermatogenesis and proposed that long term isolation of a group of individuals homozygous for a particular Robertsonian translocation chromosome could theoretically lead to the establishment of a new human subspecies with a full genetic complement in 44 chromosomes. However, to date, no such phenomenon has been reported for modern humans. In contrast to current monogamous social structure, early modern humans are thought to have lived in poly-gamous mating systems, similar to those typical for gorilla [52]. This has likely facilitated HSA2 spreading and its conversion from heterozygous to homozygous state (Fig. 2).

### likely facilitated

Out of the HSA2 fusion site directly flanking genes, *DDX11L2* expression is restricted to testis, *RPL23AP7* and *CBWD2* are expressed in the brain, and *WASH2P*, *FAM138B*, and *RABL2A* have expression in the brain and testis higher than in other organs [53, 54]. Transcription profile change of some of these genes in the brain and/or testis might have resulted, respectively, in an improved cognitive functions and/or enhanced fecundity in individuals with heterozygous and homozygous HSA2. Alternatively, given the size of the genomic material absent on HSA2 when compared with Great Apes (Fig. 1), I propose that evolution of these early humans might have been driven primarily by loss of gene(s) in the most subtelomeric regions of the ancestral chromosomes IIp and IIq. Unfortunately, gene content in these genomic regions in Great Apes is currently unknown. Gene loss and pseudogenization have been shown to be pervasive engines of genetic variation during primate speciation [55, 56]. Interestingly, compared to non-human primates, the human genome has had the fewest number of gene losses [57, 58].

## Conclusions

The formation of HSA2 may have been a critical evolutionary event influencing e.g. higher cognitive functions, upright posture, endurance running, or improved thermo-regulation by an enhanced sweating capacity in various subspecies and populations of hominins. Next generation sequencing of *Homo heidelbergensis* and *Homo erectus* genomes is technically challenging due to genomic DNA degradation and chemical modifications, but eventually may enable more precise timing of HSA2 origin. Moreover, complete DNA sequence reconstruction of the Great Ape orthologous subtelomeric chromosomes will help to better understand its evolutionary consequences. The multi- versus single-origin hypotheses can be tested by analyzing the HSA2 fusion junction in various modern human populations. Detailed studies of the genetic processes leading to brain enlargement [59] can open new perspectives for better understanding the complex and unique relationships among molecular genetics and high human mental capabilities, including social life and development of culture.

## Abbreviations

HSA2: Human chromosome 2
LCR: Low-copy repeats
Mya: Million years ago
NAHR: Nonallelic homologous recombination
NGS: Next generation sequencing
PATRR: Palindrome AT-rich repeat
PTR: Chimpanzee chromosome
ROB: Robertsonian translocation
